# The Kinematics of Trunk and Upper Extremities in One-Handed and Two-Handed Backhand Stroke

**DOI:** 10.2478/v10078-011-0071-4

**Published:** 2011-12-25

**Authors:** Adam Stępień, Tadeusz Bober, Jerzy Zawadzki

**Affiliations:** 1 University School of Physical Education in Wroclaw, Poland

**Keywords:** biomechanics, motion analysis, tennis, backhand stroke

## Abstract

The aim of this study was to present kinematics of trunk and upper extremities in tennis players who perform one-handed and two-handed backhand strokes. The study aimed to address the question of whether one of those techniques has some important advantage over the other. If so, what makes it superior?

The study included 10 tennis coaches with average coaching experience of 9 years. The coaches were asked to hit 15 one-handed and two-handed backhands. The tests were carried out in a laboratory. A sponge ball was used in order to protect the measurement equipment. Video motion analysis was carried out using BTS SMART system; images were recorded with 6 cameras with a rate of 120 frames per second. The analysis of both backhand strokes focused on the second phase of the stroke (acceleration).

The use of an eight-element model of human body for description of upper body motion in both techniques revealed kinematic differences in how both backhands are performed. The two-handed backhand was performed in closed kinetic chain with 8 degrees of freedom, whereas the one-handed backhand involved an open kinetic chain with 7 degrees of freedom. Higher rigidity of upper extremities which are connected with trunk in the two-handed backhand, contributes to an elevated trunk effect in this stroke. This is confirmed by higher component velocities for racket handle, which result from trunk rotation in the two-handed backhand and a negative separation angle in the two-handed backhand at the moment of contact of the racket with the ball.

The study does not provide a clear-cut answer to the question of advantages of one technique over the other; however, it reveals dissimilar patterns of driving the racket in both techniques, which suggests the need for extending the analysis of techniques of both backhands with additional kinematics of tennis racket in consideration of measurements of ball velocities.

## Introduction

Tennis emerged as a sport in the nineties of the 19th century. In 1874, Major W.C. Wingfield patented a new game and named it lawn tennis. The court for the game was initially in the form of two trapezoids connected with their lower bases. In 1877, it adopted a contemporary shape (rectangle). Taking a closer look at the game of tennis reveals changes which have occurred in customs, techniques and principles of the game throughout the years. Initially, the game of tennis was predominantly played by the members of upper classes, especially by those from English-speaking countries, where social status imposed a suitable dress code and behaviour in the court on a player. Practice of tennis court competition shows that contemporary tennis adopted entirely new rules of the game. The pressure on sport results, money and fame has brought about changes in both tennis technology and customs. Enormous technological advances over the last 30-40 years have caused that modern materials started to be used to manufacture rackets and balls, which partially brought a reduction in duration of rallies during tennis matches. A study by Kovacs (2004) demonstrated that the average time of a rally in US Open in 2003 was twice shorter than in 1988. Changes connected with the rally time forced players to seek new techniques of effective performance of strokes.

When playing, a tennis player, depending on the adopted tactics and actions taken by the opponent, might use a variety of tennis strokes. One of the most basic tennis strokes is the backhand, which can be performed in two ways: as a one-handed or two-handed stroke. Looking back at the eighties of the past century, it was the one-handed backhand that was used in professional tennis, both by women and men (Association of Tennis Professionals, 1980–2010; Woman Tennis Association, 1980–2010). Currently, the two-handed backhand is gaining more popularity and it becomes a secret weapon used by a number of female and male world elite players (Reid, 2001). Growing popularity of the two-handed backhand might suggest an advantage of this technique over the one-handed backhand. However, the question remains: what makes the two-handed backhand better than the one-handed stroke?

The first studies on one-handed and two-handed backhand kinematics were presented by Groppel in 1978. These studies analysed the one-handed backhand using a five-component body model, which allowed for twist of hips/trunk (around the long axis) and movements of arm, forearm and hand/racket. The analysis of the two-handed backhand was simplified, the author used a two-segment body model with rotation of hips/trunk (around the long axis) and then the movement was transferred on upper extremities (Groppel, 1978). In contemporary game of tennis, the five-element body model proposed by Groppel (1978) remains unchanged. Reid and Elliott (2002) argue that contemporary two-handed backhand should also be analysed by means of a five-element model of a tennis player’s body.

The literature emphasizes factors which have an impact on the effectiveness of backhand strokes, both one-handed and two-handed. These factors include, among other things, movement duration. The studies by Reid and Elliott (2001) demonstrated that a shorter time of performing the second phase of the strokes (driving a racket towards the ball until the contact with the ball) gives an opponent time to read the intentions of the player. Previous studies have also suggested that the two-handed backhand is more effective in concealing the stroke compared to the one-handed backhand. According to Elliott (2001), the factors which affect the speed of the stroke, confirming the effectiveness of the one-handed and two-handed backhand include: use of muscle elastic energy in the stretch-shortening cycle (SSC), range of racket displacement towards the ball, proper coordination based on ‘the principle of the chronological coordination of individual impulses’ (Hochmuth, 1984), combining translational and rotational movements. Other factors listed by Elliott (2001) included endurance, power, muscle strength and the used equipment.

The results of the studies by Reid and Elliot (2001) demonstrated that elite players could generate similar velocities of translational motion of the racket in either the one-handed or two-handed backhand. The results suggest that the velocity of the racket tip at the moment of contact with the ball does not prejudge an advantage of two-handed stroke over one-handed backhand. In order to examine the advantage of the two-handed backhand over the one-handed one, a more detailed analysis of the structure of both types of strokes should be carried out.

Previous descriptions and analyses of the two-handed backhand model have not incorporated the role of a non-dominant extremity (only the angles measured at the moment of hitting the ball were presented (Reid and Elliott, 2001)), and the quantitative analysis of contribution of translational and rotational movements of the trunk and upper extremities in the one-handed and two-handed backhand has not been carried out. Additional knowledge about both backhand techniques and replacement of the five-element model of a tennis player with higher number of elements would provide a broader image of the one-handed and two-handed backhand. It should also be emphasized that upper extremities involved in performing a stroke, depending on the method, constitute an open (one-handed backhand) or closed (two-handed backhand) kinetic chain. These chains have different structures and differ in their mobility. These differences suggest the need for a kinematic analysis, comparison and assessment of contribution of individual chain links to performing a movement, which can be also useful in obtaining additional information regarding both techniques.

## Material and methods

The experiment included 10 tennis coaches, with average body mass of 77.8 kg and body height of 1.79 m. The subjects were experienced tennis coaches, who were able to perform both backhand techniques with similar easiness. Average coaching experience among the subjects amounted to 9 years. Average competitive experience was 7 years. The subjects were asked to perform 15 flat (without rotation) backhands, both one-handed and two-handed, from a closed stance, and 10 to 15 trials performed by each person, verified in terms of correctness (hitting the target on the wall with the ball) were included in the analysis. A spongy ball was used for the investigations in order to protect the laboratory test equipment from damaging.

The tests were carried out in laboratory conditions, using BTS SMART system for a comprehensive video motion analysis. Frame rate used during this analysis amounted to 120 frames per second. Technical specifications of BTS system are presented in Table 1

The kinematic analysis was carried out in relation to a three-dimensional reference system, connected with the ground and oriented so that: motion along X axis (horizontal) was in agreement with the direction of the stroke, Y axis was vertical and Z axis was transverse.

**Figure 1:**
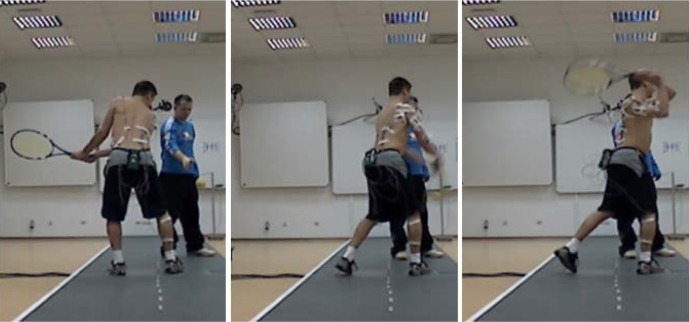
View of performed motor task (two-handed backhand stroke)

Backhand stroke was divided into three phases. Phase 1, preparation phase, began from a swing with a racket to the rear and ended at the moment of reversing the direction (defined by the sense of the vector of velocity of the racket head tip). Phase 2, acceleration, ended at the moment of contact of the racket with the ball. Phase 3, follow through, involved stopping racket movement after hitting the ball. The player began movement waiting already in a backhand stance, then a natural slight pulling back of the racket (phase 1: swing) was observed before the forward movement (Figure 1). The analysis presented in this study relates to the second phase of the stroke.

**Figure 2:**
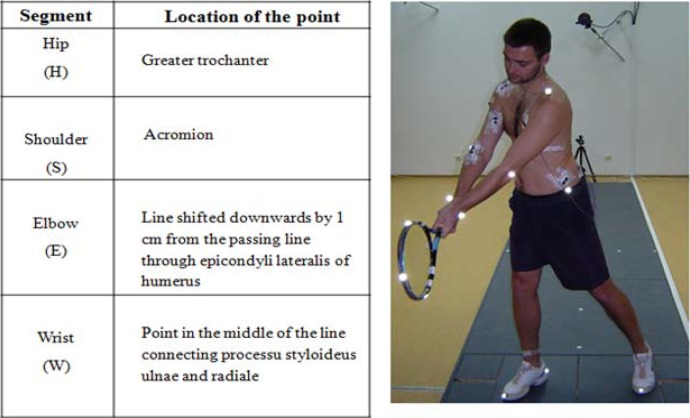
The distribution of markers on the body and tennis racket

An eight-element model of a tennis player’s upper body part was employed in the study, with spatial arrangement of body parts described by the coordinates of body points (on which the markers were placed) These points included: left and right greater trochanter in femur (H), left and right shoulder (Sh), both elbows (E) and wrists (W). Additional markers were located on the racket head tip (RT) and racket handle butt (Rh). The points were identified according to the fourteen-segment human body model. A detailed diagram of arrangement of these points is presented in Figure 2. Based on the coordinates of spatial points in the body, the numerical differentiation method was employed for determination of velocities of body parts in the study subjects.

Polar coordinates for shoulder line in horizontal plane were measured as a slope of a straight line through the shoulders (left and right) in relation to Y–Z plane of the external frame of reference. The analogous procedure was employed for the definition of the angle for hip line position. The angle of ‘twist’ of the trunk was measured as an angular difference between position of the hip and shoulder line, similarly to Reid and Elliott’s investigations (2002), who termed this difference a separation angle. It was aimed at demonstrating the difference in shoulders’ rotation in relation to hips at the moment of hitting the ball with the racket for one-handed and two-handed backhand.

## Results

**Figure 3:**
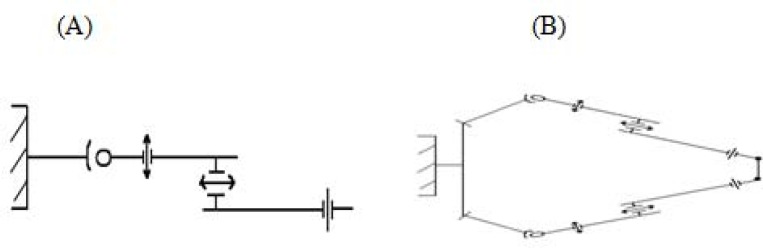
Structural diagram of the upper extremity in the one-handed backhand (open kinetic chain), with the mobility relative to the trunk, W = 7 degrees of freedom (A) and both upper extremities in the two handed backhand (closed kinetic chain), with the mobility relative to the trunk, W = 8 degrees of freedom (B)

In the literature, mobility of kinetic chains is regarded as the number of degrees of freedom of movable links in a chain in relation to an immovable base, which is the trunk in our case. The equation: $W=6n-\sum_{i=3}^{5}Pi\times i$ was used for calculation of mobility of kinetic chains of upper extremities. Upper extremity involved in one-handed stroke is identified as an open kinetic chain, i.e. a chain in a serial configuration of 7 degrees of freedom, whereas in two-handed stroke, both extremities form a closed kinetic chain with mobility of 8 degrees of freedom (Figure 3). Velocities in body parts in subjects which were observed during performing the one-handed and two-handed backhand were determined based on the distance covered by individual body parts (by means of numerical differentiation) (Knudson and Bahamonde, 2001).

**Figure 4:**
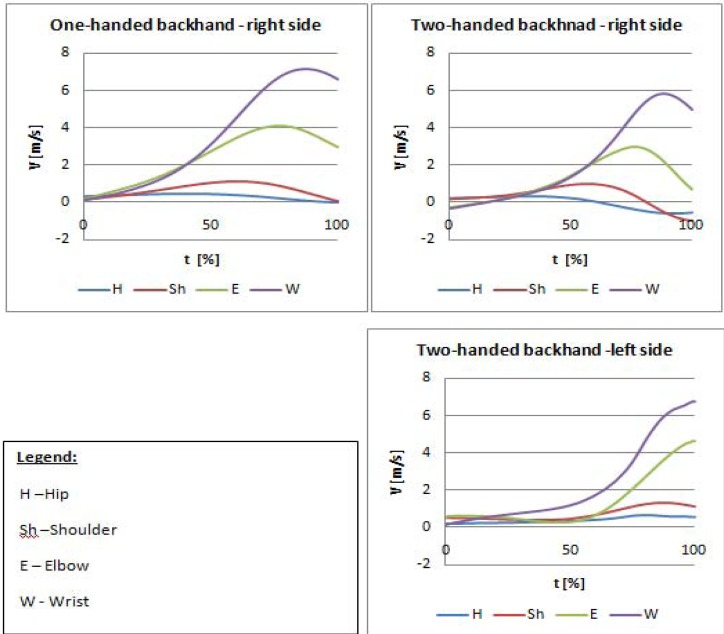
Velocity of selected parts of the body while performing a backhand. The average values of the selected coach.

Mean velocities in the points on the body during performing the one-handed and two-handed backhand (second phase of the stroke) for a selected coach are presented in Figure 4. Other subjects exhibited similar patterns of these relationships. In both types of strokes, maximal velocities observed for individual body parts increased in ascending order from hips to wrists. In the one-handed backhand, maximal velocities of body parts occurred earlier and showed higher level compared to two-handed backhand (right side of the body). In the two-handed backhand, the non-dominant side (left hip and left upper extremities in right-handed subjects) reached the maximal velocities just before or at the moment of hitting the ball with the racket; maximal velocities were observed for trunk and elbow compared to dominant extremity during the one-handed and two-handed backhand (Table 2).

**Figure 5:**
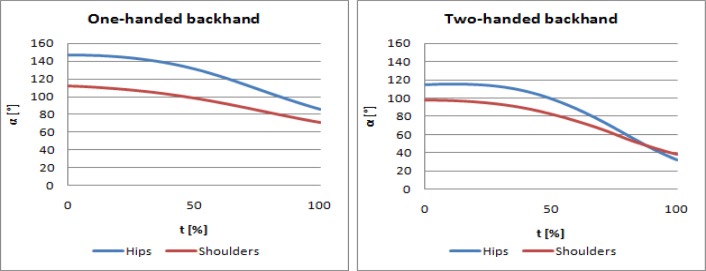
The average rotation of the hips and shoulders in the oneand two-handed backhand, registered with one of the subjects.

Figure 5 presents a mean profile of upper and lower rotation of trunk elements in the two types of strokes (one-handed and two-handed backhand) during the second phase of the stroke.

Separation angle was determined by means of subtracting the shoulder angle from the hip angle at the moment of hitting the ball. Positive results occur if the shoulder line does not cover the hip line at the moment of hitting the ball, whereas negative results mean that the shoulder line covers the hip line at the moment of contact of the racket with the ball (Reid and Elliott, 2002).

These differences are presented in Figure 5. Separation angle was employed to demonstrate different contribution of shoulder rotation in relation to hips during the one-handed and two-handed backhand. Table 3 presents the results which confirm this difference in other coaches.

**Figure 6:**
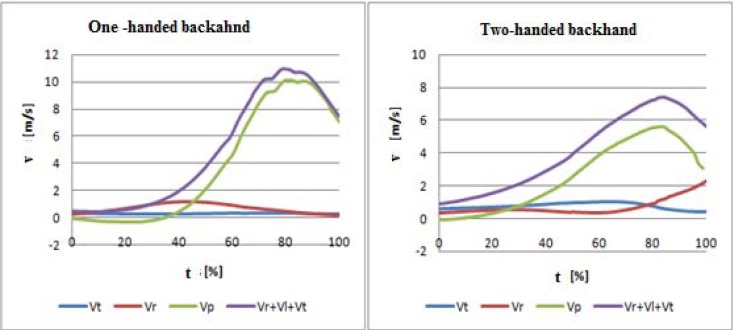
*The linear velocity components presented in the one-handed and two-handed backhand along X axis. The average data of selected coach.****V****_t_– translational velocity of upper trunk in the direction of impact;****V****_r_- translational velocity of racket handle resulting from rotational motion of trunk around the long axis;****V****_p_- translational motion velocity of racket handle in relation to trunk along X axis;****V****_t_****+ V****_r_****+ V****_p_– the sum of velocity components of the translational motion of the trunk and upper extremities*

Seeking more comprehensive information about the role of extremities in the one-handed and two-handed backhand made authors identify components of translational and rotational motion of the trunk and upper extremities in both backhand techniques. These components included: velocity of translational motion of the upper part of the trunk towards hitting the ball (v_t_), translational velocity of the racket handle resulting from rotational motion of the trunk around the long axis (v_r_) and translational motion velocity of the racket handle in relation to the trunk along X axis (v_p_). The results obtained for a selected coach are presented in Figure 6. The velocity profiles were similar in other subjects (Table 4). The velocity resulting from trunk rotation suggests higher contribution of this body part to generation of stroke velocity compared to the one-handed backhand, where translational velocities in racket handle, resulting from rotational motion of the trunk around the long axis was lower in the coaches included in the study (Table 4, Figure 6).

In other subjects, mean translational velocities of the racket handle in the two-handed backhand also exhibited higher levels (which resulted from rotational trunk motion around the long axis) compared to the one-handed stroke at the moment of racket contact with the ball in the second phase. At the same time, a different structure of performing the one-handed or two-handed backhand caused that profiles of these velocities showed a different character. The results are presented in Table 4. The profiles of (Vr) are presented in Figure 6.

## Discussion

The five-element body model of a tennis player has been widely used in scientific research regarding tennis, in the aspect of both one-handed and two-handed backhand (Bahamonde, 2005; Reid, 2001; Reid and Elliott, 2002; Reynolds, 1996). Replacement of the five-segment body model with an eight-segment one, which considers the other extremity, used in the present study, allowed for determination of the structure of mobility of kinetic chains in the one-handed and two-handed backhand. These chains have a different structure and differ in terms of mobility. The one-handed backhand forms an open kinetic chain with 7 degrees of freedom, whereas the two-handed backhand can be described as a closed kinetic chain with 8 degrees of freedom, which is characterized by higher mobility compared to the open chain typical of the one-handed backhand. However, the structure of the open chain (one-handed backhand) is composed of three elements which form three kinematic pairs, whereas the two-handed backhand, which forms a closed chain, has twice more elements and forms six kinematic pairs. With the difference of one degree of freedom, the two-handed backhand, performed in a closed kinetic chain with upper extremities connected with the trunk in a more rigid manner, ensures higher rigidity compared to the open kinetic chain typical of a one-handed backhand.

It is generally accepted that an effective stroke requires a particular coordination of movements of body parts, expressed in ‘the principle of the chronological coordination of individual impulses’ (Hochmuth, 1984; Lees, 2003; Palut and Zanone, 2005). Bober (1977) argues that maximal velocity should be reached before the ball is hit, which would result from the principle of sequential movements. The presented results for the one-handed backhand are in agreement with the principle, whereas in the two-handed backhand this applies only to the right extremity (Figure 4). This might be attributed to different roles performed by upper extremities in a two-handed backhand. Maximal velocities and their order suggest that the left extremity (in right-handed players) plays a role of a dominant extremity, generates velocity in X axis, whereas the right extremity performs the role of an auxiliary extremity which stabilized the left extremity during the stroke. In the one-handed backhand, both functions are taken over by one extremity. It should be also emphasized that mean maximal wrist velocities of the coaches included in the study were obtained for the dominant extremity in the one-handed backhand stroke, however, at the moment of hitting the ball with the racket, higher velocities were observed in the non-dominant extremity during the two-handed backhand. The results of investigations of kinematics of tennis racket presented in the study by Stępień and Bober (2009) demonstrated different types of drive between one-handed and two-handed backhands. Previous biomechanical descriptions and analyses of a two-handed backhand did not take into consideration the role of the non-dominant extremity.

In a two-handed backhand, which forms a closed kinetic chain, the motion of hands which hold the racket is composed of a translational motion of the upper part of the trunk (toward the target), movement of extremities caused by trunk rotation around the long axis and translational motion resulting from displacement of the extremities in relation to the trunk in a frontal plane. Because of rotational motion of the trunk at the angular velocity ω, a racket handle reaches linear velocity v_r_=ω·R, with R being a radius measured from the axis of trunk rotation to racket handle. Furthermore, racket velocity can be increased by adding translational motion of palms which hold the racket handle in relation to the body trunk. This is possible only if upper extremities are bent in elbows. Velocity of this motion, v_p_ is added to the velocity v_r_ and v_t_.. The results (Table 4) and profiles presented in Figure 6 reveal that using the properties of a closed chain causes that trunk rotation affects component velocity of the racket handle along the X axis in a two-handed stroke more than in a one-handed backhand. Similar results for hip and shoulder rotation in the 2nd phase of the stroke in a one-handed and two-handed backhand were obtained by Reid and Elliott (2002), who demonstrated that hip rotation in a one-handed and two-handed backhand was similar and shoulder rotation was higher in a two-handed backhand, which suggests the importance of rotational and twisting motion of the trunk in this type of a stroke. In the two-handed backhand, the line of shoulder arrangement rotated beyond the line of hips, which generated negative values of the separation angle. Shoulders in tennis players who preferred the one-handed backhand did not rotate beyond the hip line, which caused that this angle was positive, both in the investigations carried out by the authors cited above and the authors of the present study.

Schönborn (1998) argues that coordination abilities of performing the movement might be the most important factor in the process of learning the movement. It is generally accepted that a two-handed backhand requires less effort compared to the stroke performed with one extremity. It is also the main reason for choosing a two-handed backhand in tennis coaching by the overwhelming majority of coaches. Reid (2001) argued that coaches should demonstrate knowledge which allows them to adjust backhand individually in each player and to emphasize their physical characteristics, coordination and style.

Kinematics of upper extremities and the trunk in the one-handed and two-handed backhand supported with an eight-element model of a tennis player body, does not entirely answer the question of the advantage of one type of the stroke over the other. However, it can be concluded, that the two-handed backhand, with its closed kinetic chain, is characterized by the other structure and mobility compared to the chain which was formed in the one-handed backhand. Use of an eight-element tennis player body model allowed the authors of the present study to identify the role of the extremities in the two-handed stroke. The obtained results of component velocities in the racket handle, resulting from trunk rotational motion along the long axis and the velocities of the left side of the body (in right-handed subjects) confirmed greater role of trunk rotation in the two-handed backhand compared to the one-handed stroke. The results for the one-handed backhand confirmed previous reports which demonstrated that particular body part velocities were generated according to the principle of summation of partial impulses.

## Conclusions

1. The two-handed backhand is performed within a closed kinetic chain with the mobility higher by one degree of freedom compared to the one-handed backhand, performed in an open kinetic chain. However, use of both upper extremities in the two-handed backhand ensures higher rigidity through the connection of the extremities with the trunk compared to a single upper extremity in the one-handed backhand. This opens up opportunities for enhanced contribution of kinetic energy of the trunk to performing a stroke.
2. In the two-handed backhand, left body side (right-handed subjects) in the coaches included in the study reached higher velocity at the moment of hitting the ball with the racket compared to the right side in the one-handed stroke. The results of the studies by Reid and Elliot (2001) demonstrated that elite players are able to generate similar translational motion velocity of the racket either in the one-handed or two-handed backhand, which suggests different type of drive.
3. Upper extremities perform different functions in the two-handed backhand: left extremity generates velocity in the racket, whereas the right one performs the role of a supporting extremity which controls the right one (in right-handed player). The one-handed backhand cannot be supported in this manner, and the lack of this function might suggest a higher load in the muscles compared to the two-handed backhand.
4. It would be useful to extend the analysis to incorporate the 1st and 2nd phase of the stroke in one-handed and two-handed backhands, including kinematics of a tennis racket in consideration of measurements of ball velocity, which would provide additional information on both types of backhand.

## Figures and Tables

**Table 1:** Specifications of the system for the motion analysis (BTS)

**Cameras:**	6 digital cameras, powered by a central processing unit
**Acquisition frequency:**	120 frames per second
**Resolution:**	1,4 Megapixels
**Data transmission:**	GIGABIT Ethernet
**Markers:**	Passive, reflects IR, 15mm
**System for markers recognation:**	Identifying elements of a “blob” in the gray scale (areas with similar intensities)

**Table 2:** Averaged maximum velocity and the relative time of occurrence for the analyzed number of repeats obtained in the one- and two-handed backhand by coaches in the second phase of the stroke (acceleration phase)

		Location of the points			
Type of stroke / extremitie		Hip (H)	Shoulder (S)	Elbow (E)	Wrist (W)
One-handed backhand / dominant	v_max_ [m/s]	**0,45**	**0,90**	**3,77**	**7,52**
	t [%]	8	50	70	82
Two-handed backhand / dominant	v_max_ [m/s]	**0,37**	**1,08**	**3,30**	**6,31**
	t [%]	24	55	76	87
Two-handed backhand / non dominant	v_max_ [m/s]	**0,67**	**1,41**	**4,86**	**6,85**
	t [%]	96	100	100	100

**Table 3:** „Separation angle” in the moment of impact with the ball in the one-handed and two handed backhand

**Subjects**	**Separation angle [°]**	
	One-handed backhand	Two-handed backhand
1	14,37	−6,08
2	21,13	−1,09
3	18,58	−9,39
4	1,63	−9,92
5	8,06	−9,47
6	3,42	−7,95
7	1,07	−13,15
8	11,92	−0,67
9	10,35	−1,23
10	2,06	−5,79
Mean	**9,26**	−**6,47**
SD	**7,26**	**4,31**

**Table 4:** Averaged translational velocity of the racket handle (for the analyzed number of repetitions from 10 to 15) due to rotation of the trunk at the time of racket contact with the ball in the second phase of the stroke in the one-handed and two-handed backhand.

Subjects	One-handed backhand V_r_ [m/s]	Two-handed backhand V_r_ [m/s]
1	1,58	1,65
2	0,16	1,83
3	0,13	2,28
4	0,79	2,39
5	1,38	2,79
6	0,17	1,68
7	1,76	2,64
8	1,09	1,19
9	1,38	2,01
10	0,96	2,42
Mean	**0,94**	**2,09**
SD	**0,61**	**0,50**
